# Advances in light manipulation in greenhouse horticulture: the innovative smart covers

**DOI:** 10.3389/fpls.2025.1640530

**Published:** 2025-10-07

**Authors:** Farzaneh Zamani, Luigi Giuseppe Duri, Mauro Mori, Roberta Paradiso

**Affiliations:** ^1^ Department of Agricultural Sciences, University of Naples Federico II, Naples, Italy; ^2^ Department of Agriculture, Food, Natural Resources and Engineering (DAFNE), University of Foggia, Foggia, Italy

**Keywords:** diffusive covers, photoselective covers, luminescent covers, switchable covers, photosynthesis, temperature regulation, energy efficiency

## Abstract

Greenhouses play a key role in modern agriculture by creating controlled environments to fulfil specific plant climatic requirements, allowing the extension of the growing season and improving the crop productivity and product quality. Light, in terms of quantity (intensity), quality (spectral composition), and duration (photoperiod), is a crucial factor in driving plant performance in protected cultivation. Solar radiation is significantly affected by the greenhouse framework and cover material. The use of smart materials, including diffusive, photoselective, luminescent, and switchable covers, can positively modify the light intensity, spectrum, and distribution, improving the greenhouse light environment, hence the plant growth, morphology, and metabolism. This review summarizes the state of art of research on innovative covers suitable for modern greenhouse horticulture and their effects on plant performance in vegetable and ornamental crops.

## 1 Introduction

**Light** is essential in agriculture, driving both the plant photosynthesis and photomorphogenesis. Indeed, while photosynthesis enables plants to convert light into chemical energy, supporting growth and productivity, photomorphogenesis encompasses crucial developmental, morphological and metabolic changes in response to light stimuli, such as leaf expansion, stem elongation, flowering, and biosynthesis of antioxidant compounds (Paradiso and Proietti, 2022). These processes are interconnected, allowing plants to optimize the light energy utilization and the adaptation to the growth environment, and can be strategically harnessed in controlled horticulture.

Among the three main parameters of light, namely the intensity, spectral composition and photoperiod, light spectrum has been gaining increasing attention in the last years, as the knowledge of plant response to the different wavebands strongly increased, also thank to the use of light emitting diodes (LEDs) in plant research.

Plants perceive the light spectral composition through 5 distinct classes of specific photoreceptors, with a high sensitivity for the different wavebands even at very low light intensity (Paradiso and Proietti, 2022). In the visible range of light radiation, these wavebands correspond to different colors: blue (B, 445–500 nm), green (G, 500–580 nm), yellow (Y, 580–600 nm), orange (O, 600–620 nm), red (R, 620–700 nm), and far red (FR, 700–775 nm). Photoreceptors of the phytochrome family absorb R wavelengths; three different photoreceptors, cryptochromes, phototropins, and the ZTL/FKF1/LKP2 complex, perceive B and UV-A wavelengths; the UVR8 photoreceptor is sensitive to ultraviolet (UV) radiation, particularly UV-A (315–380 nm) and UV-B (280–315 nm). The light sensing machinery is very sophisticated, often involves the plant hormonal signaling pathways, and concerns numerous processes driven by light colors, in the complex phenomenon of photomorphogenesis (Paradiso and Proietti, 2022).

It is now known that R and B are the most efficient wavelengths in sustaining photosynthesis, driving the electron transport and rubisco activity (Liu and Van Iersel, 2021). The R light promotes plant growth, flowering and fruit production (Zhang et al., 2020), while B influences the leaf expansion and stem elongation and regulates stomatal opening. Together with R, FR can induce reproduction and trigger morphological changes to optimize the light capture in shade conditions, through shade avoidance mechanisms activated by low R-FR ratios. Additionally, B and UV stimulate the biosynthesis of antioxidants, to enhance the plant tolerance to stress conditions, eventually improving the produce quality (Rai, 2020; Jaiswal et al., 2021). The G light, alongside R and B, plays a key role in the assimilation process, penetrating deeper into the plant canopy and leaf tissues, hence supporting photosynthesis in the inner plant and leaf layers, where R and B are less effective. Moreover, G modulates some physiological processes, such as the stomatal opening, and morphological responses, like shade avoidance, complementing the R and B action (Paradiso et al., 2025).

The global adoption of **protected cultivation** has grown substantially and, according to recent estimates, vegetable greenhouse production covers more than 500,000 hectares, with a significant portion (90%) adopting plastic covers (Chavan et al., 2022). Glasshouses make up approximately 20% of the total surface area and are mainly located in regions, such as Europe (Baeza and López, 2012), where diverse climates present different challenges. For instance, Northern regions face low light intensity and temperature, and short photoperiod in winter, while Southern areas, particularly in Mediterranean basin, experience high radiation and heat (Von Elsner et al., 2000). Most other greenhouse structures are covered with plastic materials, often complemented by shading nets. These are more common in regions like Asia and North America, where cost-effective solutions are favored, and offer flexibility and efficiency, balancing light transmission with insulation (Maraveas, 2019).

Managing **light in protected cultivation** is critical for optimizing the production schedule and the crop yield. Innovative technologies such as diffusive or photoselective materials have been shown to increase crop productivity by enhancing light spectrum and distribution, also reducing heat stress and optimizing photosynthesis. Light-diffusing materials, for example, can increase crop productivity by 3% to 30% depending on the crop and growing conditions (Zhang et al., 2024a). With advancements in smart covers and light manipulation technologies, the efficiency of greenhouse farming continues to rise, reducing the energy consumption while improving the produce yield and quality (Shi et al., 2024).

Within the wavelength interval of photosynthetically active radiation (PAR), the **intensity** of light directly affects the biomass accumulation, hence covers that control the fraction of radiation entering in the greenhouse through reflection or diffusion can regulate the amount received by the crop (Romero et al., 2018). Low light intensity, like that occurring in winter cultivations, can reduce biomass production and yield (Zhang et al., 2024a). In this respect, light-diffusing covers, enhancing the light distribution in greenhouse at the canopy level, have been shown to improve the plant photosynthetic efficiency and the crop productivity. Diffusive plastic films increase the light transmittance and scatter light more evenly across the canopy, reducing photoinhibition in the upper canopy while increasing the amount of light energy reaching lower leaves (Moreno-Teruel et al., 2021). Plants grown under diffusive covers exhibit more uniform growth, higher yield, and better produce quality, with a limited energy input thank to the reduced supplementary lighting (Gattuso and Mazzola, 2023). Studies have also shown that altering **spectral composition** of solar radiation in greenhouses using photoselective materials, for example modifying the R-FR ratio (Schettini and Vox, 2010), can improve plant health and increase the crop productive performance (Lamnatou and Chemisana, 2013).

As climate change continues to alter global weather patterns, managing temperature and light in greenhouse becomes increasingly important for ensuring stable crop yields and product quality (Gruda et al., 2019). In this respect, photoselective covers can filter specific light wavelengths, helping to mitigate the temperature fluctuations, hence enhancing the plant photosynthesis and water-use efficiency, while improving the plant metabolism by boosting the biosynthesis of functional compounds (Mormile et al., 2019). Greenhouse covers that incorporate heat-controlling agents, such as those with near-infrared (NIR) reflective properties, can help mitigate high temperatures by reducing both the external heat entering the greenhouse and the internal heat generated by the absorption and re-radiation of infrared (IR) radiation from the soil. For example, in arid regions (e.g., Ishikawa in Japan), reflective films have been shown to reduce internal temperatures up to 9 °C, allowing plants to maintain a healthy growth during extreme heat events (Murakami et al., 2017).

Given the increasing importance of optimizing lighting strategies to improve crop performance and greenhouse sustainability, this study aimed at critically reviewing how the light environment can be passively manipulated through innovative smart covers. Specifically, we examined how these materials influence light intensity, spectral composition, and uniformity of distribution, driving plant growth and productivity and produce quality, in controlled environment agriculture.

## 2 Method applied for the literature review

A comprehensive review of scientific literature was conducted using the Scopus and Google Scholar databases (last update December 2024), by using the following keywords: smart greenhouse covers, innovative greenhouse covers, diffusive covers, photoselective covers, switchable covers, shading nets. A total of 96 papers were analyzed, comprising 42 review articles and 54 research articles. Among the 54 research articles, some addressed multiple types of greenhouses covers. Specifically, 26 focused on diffusive and/or reflective covers, 34 on photo-selective covers, 10 on luminescent covers, and 7 on switchable covers, with overlaps among the different categories.

These studies were predominantly related to greenhouse experiments and encompassed a wide range of crops. Specifically, 10 articles focused on leafy vegetables, 34 on fruit vegetables, 5 on fruit and small trees, and 2 on ornamental and flower species, 2 on grains and 1 on *Arabidopsis*. Among these, tomato is the most studied crop, followed by cucumber, and lettuce. Other crops, including eggplant, melon, wild rocket, and cabbage, were less represented.

**Figure 1:**
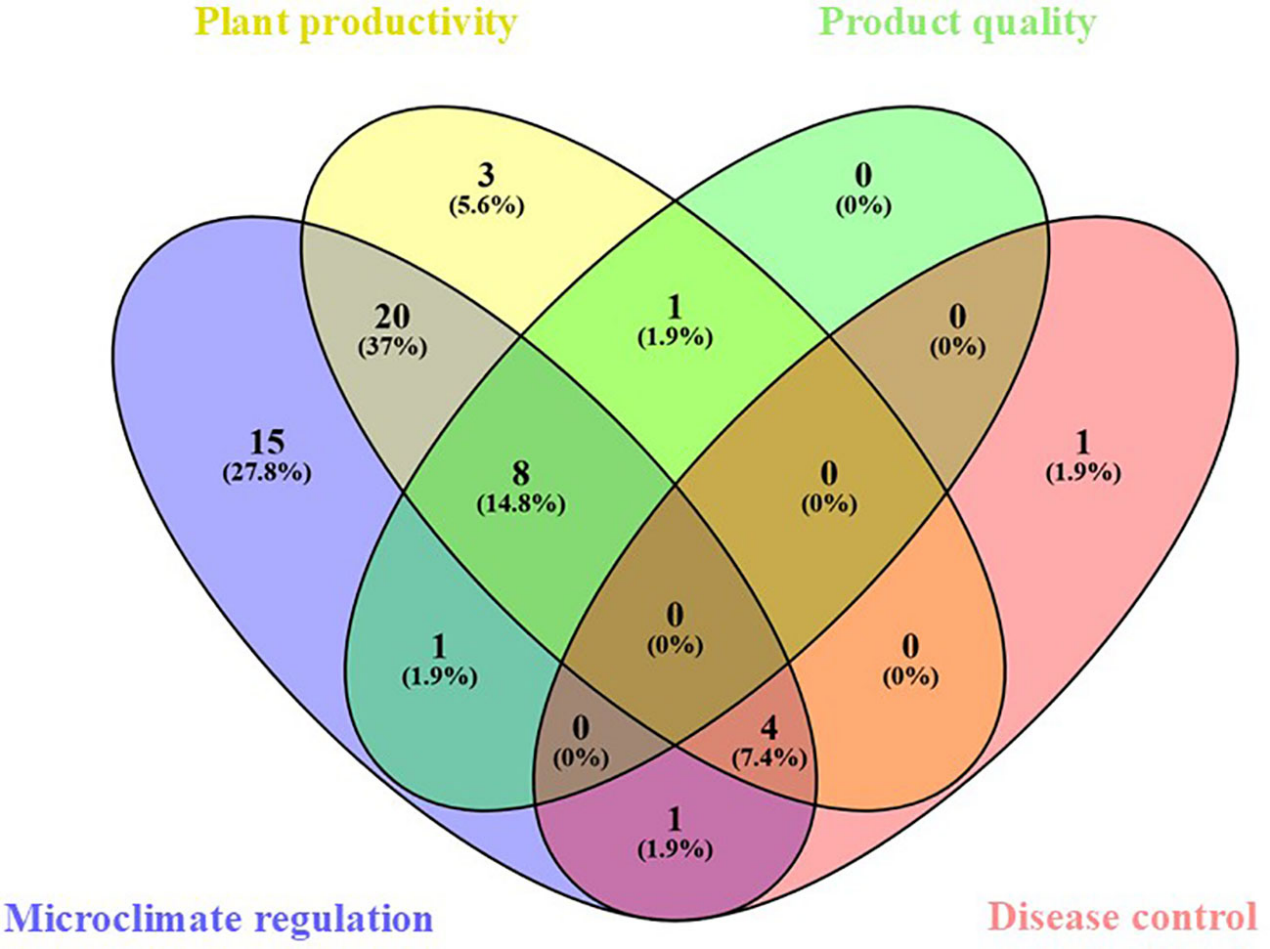
Venn diagram representing the classification of the 54 research articles collected through the literature review based on the main topics. The distribution in each group is reported as both percentage of the total and number of papers. The diagram was created using the Venny 2.1.0 software (Oliveros J.C., 2007-2015; https://bioinfogp.cnb.csic.es/tools/venny/index.html). Four different colors were assigned to the 4 main categories, and papers were grouped depending on their belonging to a single category or multiple categories, creating subsets derived from the overlap of the main categories.

The 54 research articles were further categorized into major thematic groups based on their content and focus: Microclimate regulation (49 papers), Crop yield and product quality (39 papers), Plant stress response (5 papers), Light manipulation effects on plant physiology (10 papers), Plant health (6 papers). A Venn diagram illustrating this classification is shown in **Figure 1**. The number within each section indicate the sum of studies in each category, along with their corresponding percentage on the total. The diagram was generated using Venny 2.1.0 software (Oliveros J.C., 2007-2015; https://bioinfogp.cnb.csic.es/tools/venny/index.html). Four key themes - Microclimate Regulation, Plant Productivity, Product Quality, and Disease Control - are represented by distinct colors. Studies addressing multiple topics are in the overlapping sections, where the colors merge to indicate their shared focus.

## 3 Traditional greenhouse covers

Greenhouse covers are traditionally made in glass, fiberglass and plastics, such as polyethylene (PE), polyvinyl chloride (PVC), polycarbonate (PC), and polyhydroxyalkanoates (PHA) (Maraveas, 2019). These plastic materials are widely used due to their cost-effectiveness, ease of installation, and ability to transmit light (Maraveas et al., 2023b). However, while traditional materials are effective for basic greenhouse operations, they show several limitations, including thermal insulation issues, UV-induced degradation, IR blocking, condensation buildup, dust accumulation, mechanical fragility, and environmental impact (Maraveas et al., 2023b).

**Figure 2:**
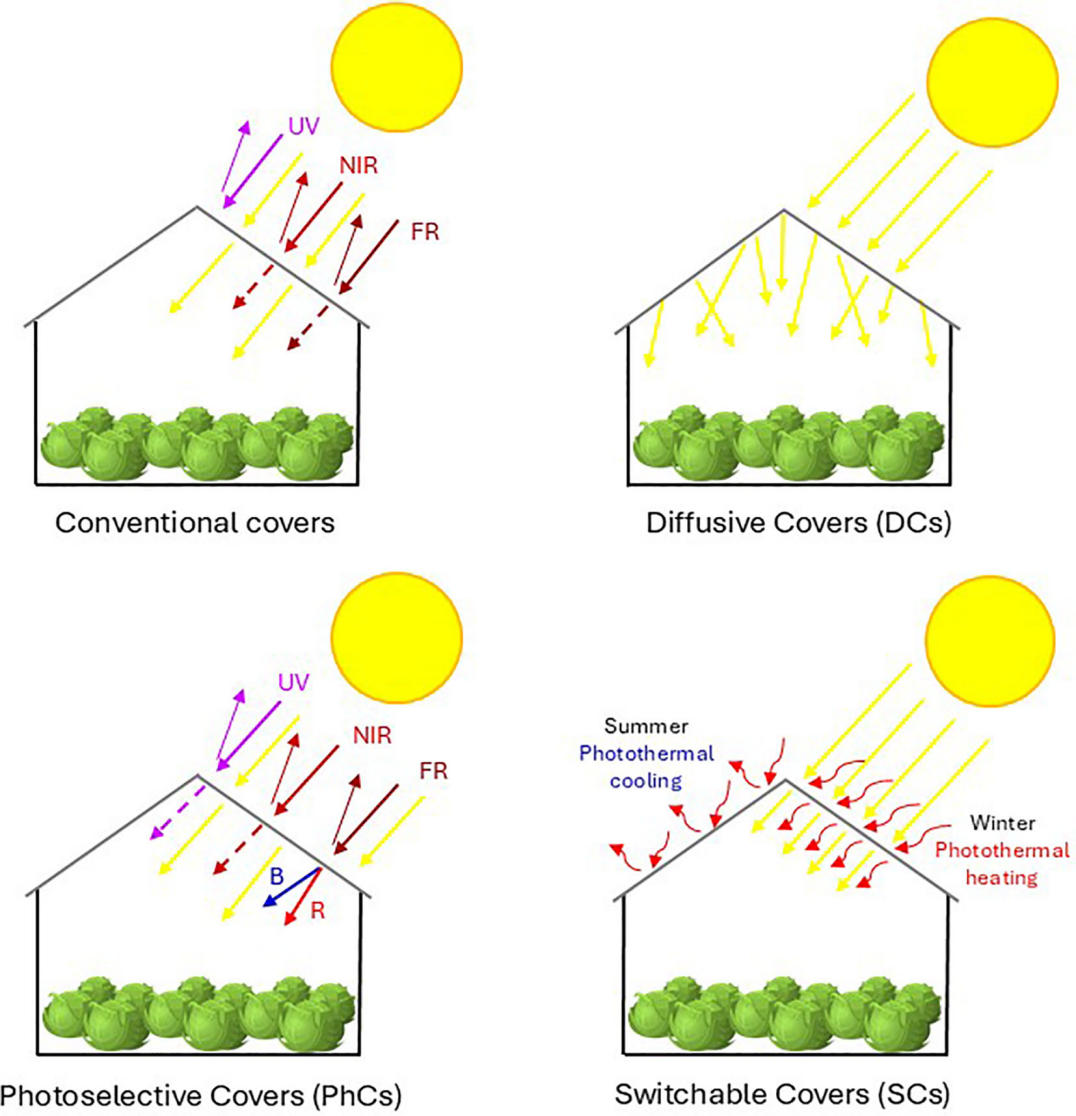
Schematic representation of light behavior across the different greenhouse cover types. Conventional covers allow the partial transmission of NIR and FR while reflect UV radiation. Diffusive covers (DCs) scatter the incoming solar radiation more uniformly across the canopy, with no effect on light spectrum. Photoselective covers (PhCs) selectively filter the different wavebands, transmitting or reflecting specific wavelengths (e.g., UV, NIR, FR) or switch them to obtain specific spectral changes (e.g., enrichment in B or R). Switchable covers (SCs) dynamically modulate light and heat transmission in response to seasonal climatic changes, providing photothermal cooling in summer and photothermal heating in winter. Solid arrows represent the full light intensity; thin arrows represent the portion of reflected light; dashed arrows indicate the portion of transmitted light; red wavy arrows represent re-radiated or reflected energy.

**Figure 3:**
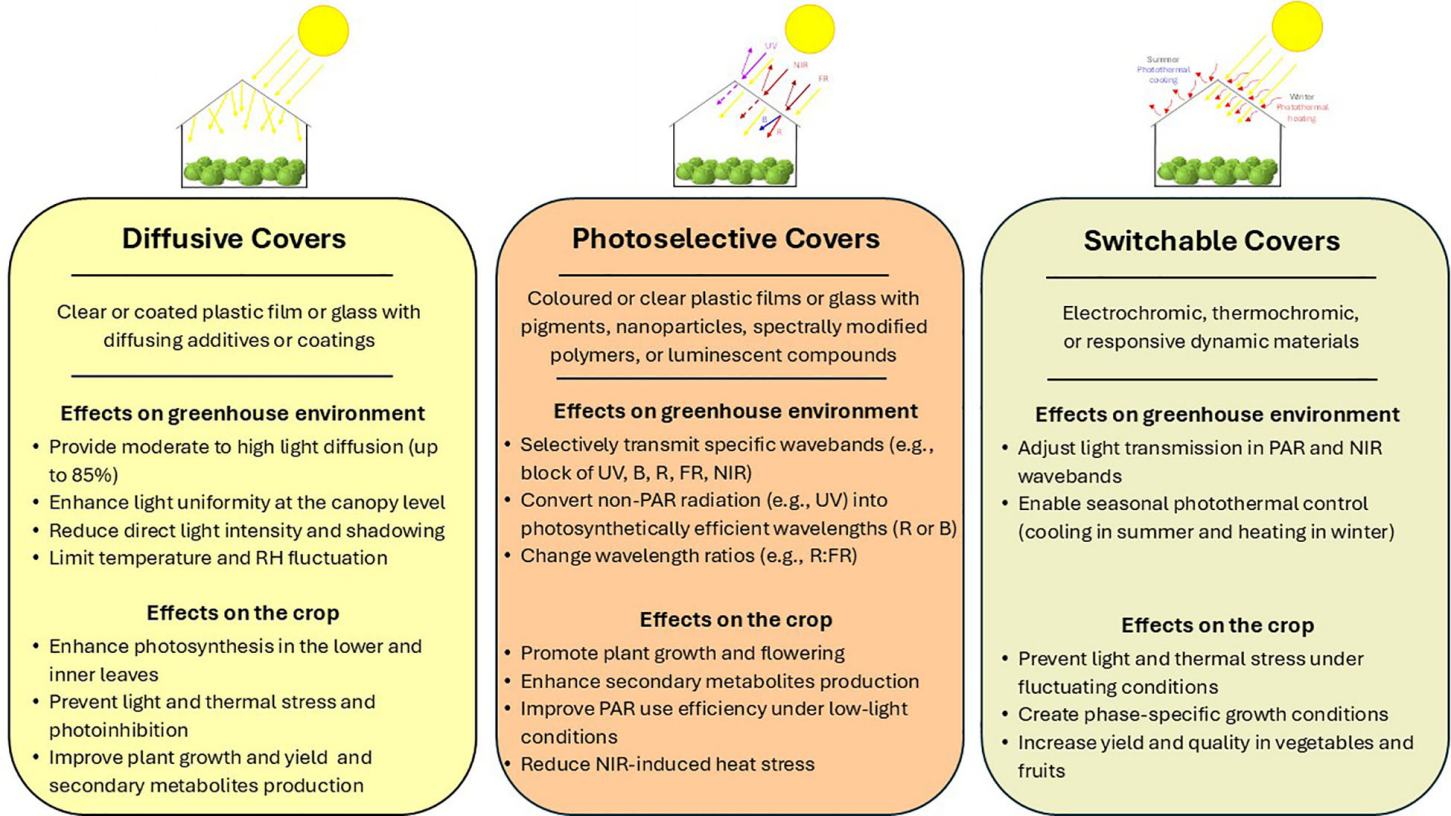
Material properties and effects on greenhouse environment and plant behavior of the different innovative greenhouse covers.

The main properties of conventional covers are summarized in **Figures 2** and **3**. Due to its strong PAR transmission (up to 90%) and NIR reflectance, which lowers the greenhouse’s energy balance, **glass** is recommended as a cladding material. In contrast, plastic polymers have a generally higher NIR transmission (Ghani et al., 2019). Consequently, glass structures are required to house IR- (and temperature-) sensitive plants. However, glass covers, known for their durability, are nonetheless fragile, heavy, and costly to install. They block certain UV wavebands, essential for pigment formation, impacting plant development negatively (McCartney, 2017). Zhang et al. (2024b) highlighted the potential benefits of glass covers, which, when oriented East-West (E-W), can enhance thermal efficiency and photosynthesis, improving yield. However, the study also noted that glass covers can increase sensitivity to diseases, as they make plants more susceptible to *Botrytis* spp (Dueck et al., 2012). **Fiberglass** has a lower optical transparency and light transmission despite the better mechanical properties (Ghani et al., 2019). The **PE** is less expensive but has a limited lifespan, usually degrading within a few years due to UV exposure, which deteriorates its structure, worsening mechanical properties and reducing light transmittance, with a consequent negative impact on the crop growth (Castilla, 2013). The **PC** is more durable and UV-resistant compared to other plastics, but it still suffers from discoloration and gradual degradation over time, with an approximate lifespan of 8 to 10 years under greenhouse conditions (Montero et al., 2011).

Another limitation of traditional covers is thermal insulation. A single layer of PE or glass offers poor insulation, raising the heating costs during cold seasons. Though double-layer PE improves thermal insulation, it reduces light transmission (by approximately 10-15%) compared to single-layer films, because of the higher absorption and scattering. This reduction affects the PAR spectrum uniformly, potentially limiting plant growth under low-light conditions (Boulard et al., 1996). Besides, the increase of the PE quantity used in double layer covers has a negative environmental impact. By an economical point of view, the frequent replacement of PE can increase the long-term expenses despite the lower initial cost, while higher-cost materials like glass and PC, more efficient in insulation, can reduce heating and cooling expenses over time (López-Marin et al., 2019). In harsh climates, the maintenance and replacement of greenhouse covers imply significant costs, prompting some operators to opt for cheaper, lower quality materials, which may reduce crop yields and increase expenditures in the long term, while worsening the greenhouse related pollution (Tanny, 2013).

## 4 Innovative smart covers

Unlike traditional covers, which primarily focus on physical durability and light transmission, smart covers incorporate technologies to control the greenhouse microclimate actively. These covers adjust parameters like light intensity, direction and spectrum, as well as thermal insulation, according to the specific crop needs, promoting the plant performance and optimizing the resource use efficiency.

### 4.1 Diffusive covers (DCs)

The main properties of diffusive covers are summarized in **Figures 2** and **3**. Diffusive covers scatter the incoming sunlight, spreading it more evenly across the greenhouse space compared to traditional clear covers. Light diffusion is achieved through the cover material itself or a specific coating, that alters the angle of incident rays, reducing the direct light intensity while minimizing the shadow areas (Hemming et al., 2016). This mechanism ensures that light penetrates deeper into the crop canopy, reaching lower and inner leaves, which otherwise would be shaded from the direct exposure (Shi et al., 2024). The DCs can increase the light uniformity index up to 20% compared to traditional covers, supporting a more uniform assimilation in the canopy profile and leading to a more balanced growth and higher yield (Moulton et al., 2020). Besides, diffusive light reduces stress conditions due to excessive radiation and photoinhibition, hence the plant need for photoprotective responses, allowing plants to better adapt to variable sunlight intensity (Paradiso et al., 2024).

By reducing direct sunlight, DCs also enhance the greenhouse microclimate by lowering temperature and increasing air relative humidity (RH). Applications in arid climate (e.g., Saudi Arabia) demonstrated that DCs can gain 77% diffuse radiation in the greenhouse in the warmest hours of sunny days, while slightly reducing the air temperature and maintaining a more constant RH level (Al-Helal et al., 2020). These covers increase the light use efficiency at the whole crop level up to 30%, leading to higher production with lower energy input (Moreno-Teruel et al., 2022). Specifically, reflective-diffusive films (RDCs), which also reflect a portion of NIR radiation while diffusing the visible portion of solar radiation, increase diffuse light by 85% compared to the corresponding traditional covers (with the same PE), reaching a diffusion percentage of 33% of the transmitted radiation. In the tested experimental conditions, they performed similarly to diffusive film (DF) in terms of temperature reduction and RH fluctuation (Al-Helal et al., 2020).

Evidence about the impact of diffusive covers on various horticultural crops are shown in **Table 1** and summarized hereinafter, based on data available in literature concerning leaf, sprout and fruit vegetables, as well as some ornamental crops.

**Table 1:** Effects of diffusive covers (DCs) on plant growth, photosynthesis and secondary metabolites content in vegetable and ornamental crops.

Species	Cultivar	Material	Cover characteristic	Effects on plant growth, photosynthesis, secondary metabolites		References
				Increase	Decrease	
*Asparagus officinalis* L.	Tainan	PE	12 % PAR diffusion, NIR-reflective, reducing heat	Photosynthesis, transpiration rate, nutrient uptake, yield (+31.4%), mineral content (e.g., Ca and Mg)	Crude fiber	Chen and Shen (2022)
*Capsicum annuum* L.	Bell Boy	PE	74.7% PAR transmission, natural ventilation, cooling through misting systems	Photosynthesis, RH	Transpiration rate	McCartney (2017)
	Bemol RZ F1	PE	55-60% diffusion, 85-90% PAR transmission	Yield (+3.1%), higher weight and fruit size	Infection of powdery mildew	Ávalos-Sánchez et al. (2023)
*Cucumis sativus* L.	Sovana F1	PE, EVA	40% diffusion, 78-80% PAR transmission, NIR-reflective	Yield (+24%), stem length, leaf area	Water and energy consumption	Alsadon et al. (2016)
	Sovana F1	PE	40-60% diffusion,75-88% PAR transmission, NIR-reflective	Yield (+20-22%), number of fruits (per m^2^), fruit fresh and dry weight		Al-Madani et al. (2024)
*Diplotaxis tenuifolia* L.	Reset	PE	58% diffusion, 90% PAR transmission, 30% UV-B transmission	Yield (+36.5), K, Ca, Mg, Na, chlorophyll, carotenoids, phenolics, antioxidant activity (HAA, ABTS)	S, ascorbic acid	Paradiso et al. (2023)
	Nature	PE	Shading nets: 50% and 79% light extinction, PE: light transmission and insulation properties	Yield (50% shading), hydrophilic antioxidant activity, ascorbic acid, minerals (e.g., K, P, Ca, Mg)	Yield as both leaf number and dry weight (79% shading)	Caruso et al. (2020)
*Lactuca sativa* L.	Longifolia Lam. Crimor-INTA	PE	93% light transmission	Dry weight, stomatal conductance, Ca distribution	Tipburn symptoms	Bárcena et al. (2019)
	Princess	PE	Uniform light distribution and reduced shading	Yield (+22%), chlorophylls, carotenoids, ascorbic acid, antioxidant activity (LAA, HAA)		Cozzolino et al. (2020)
*Solanum lycopersicum* L.	Black Cherry, Brandy Sweet Plum, Cuban Yellow, Esterina Hybrid F1, Favorito F1	PE, PC	PE: 28% direct transmission and 62% diffusion. PC: 100% diffusion	Plant growth, fruit yield, phenolics, lycopene, lutein		Ahmadi et al. (2018)
	Cherry	PE	80% transmission	WUE	Air temperature and heat stress	Hassanien et al. (2018)
	HMC44698 F1	PE	55-60% diffusion, 85-90% transmission	Yield (+3.2%), photosynthesis, gas exchange, WUE, fruit weight	Leaf temperature, heat stress	Moreno-Teruel et al. (2021)
	Ramyle RZ F1	PE	55-60% diffusion, 85–90 PAR transmission	Yield (+4.2%), fruit weight, firmness, soluble solid	Infection of powdery mildew and blight	Ávalos-Sánchez et al. (2023)
	Sakura	PE	58% light scattering, 90% transmission, 30% UV-B transmission	Yield (from 20% to 48% depending on the nitrogen rate), lycopene, carotenoids, phenolics, ascorbic acid, antioxidant activity, nitrogen use efficiency	Nitrogen, direct sunlight stress	Paradiso et al. (2024)
	Shennong	Glass	E-W orientation rather than N-S, light interception enhancement and thermal efficiency	Photosynthetic efficiency, yield	Shading effects	Zhang et al. (2024b)
	Zayda	PE	25-30% solar radiation reduction	Plant height, WUE	Heat stress, *Tuta absoluta* infestation	Ezzaeria et al. (2018)
	Komeet	Glass	50% diffusion, 78-79% transmission	Yield (+7-11%), photosynthesis, dry matter	Sensitivity to *Botrytis* spp.	Dueck et al. (2012)
	Marenza	PC	50% PAR diffusion, 85% light transmission, 8% FR transmission	Yield (+8%), photosynthesis		Moreno-Teruel et al. (2022)
*Solanum melongena* L.	Valentine F1	PE	IR absorption, UV stabilized	Plant height, stem diameter, NAR, LAR, SLA		Cemek et al. (2005)
*Valerianella locusta* L.	Princess	PE	High light diffusion and transmission	Yield (+22.4%), SPAD index, total ascorbic acid, nitrate		Cozzolino et al. (2020)
*Chrysanthemum × morifolium* L.	Midnight Time	PE	Light diffusion, higher FR light transmission	Plant growth, leaf area, shoot dry weight, number of lateral shoots, photosynthetic efficiency		Markvart et al. (2010)

The increasing or decreasing effects and the related percentage refer to results obtained in DCs compared to the corresponding clear covers.

In **asparagus** (*Asparagus officinalis* L.), the comparison between a NIR-reflective diffusive coating and a traditional PE film revealed that both covers increased the heat accumulation compared to outside but, additionally, the NIR-reflective diffusive cover provided a more uniform light distribution, improving photosynthetic efficiency in the lower canopy. This led to higher spears yield and quality, with increased calcium and magnesium content. The NIR-reflective diffusive coating also promoted a more vigorous shoot emergence after mother stalk removal, suggesting its potential in enhancing long-term stem vegetables productivity in greenhouse (Chen and Shen, 2022).

Diffusive covers enhanced the plant growth and fruit production of **pepper** (*Capsicum annuum* L.) in a Natural Ventilation Augmented Cooling (NVAC) greenhouse in Quebec (Canada) (McCartney, 2017). In this experiment, the improved light diffusion and moderated temperature led to a 28% increase in photosynthetic rate and a 31% reduction in transpiration, ultimately benefiting plant growth. Similarly, diffusive PE increased the leaf area, hence the photosynthetically active surface and assimilation capacity, which contributed to improved growth and productivity of pepper in a Mediterranean greenhouse in Southern Europe, leading to a higher marketable fruits yield (+3.1% compared to commercial PE) (Ávalos-Sánchez et al., 2023).

The use of diffusive PE films increased the leaf area and shoot elongation in greenhouse **cucumber** (*Cucumis sativus* L.) grown under arid conditions in middle east and north Africa, leading to higher biomass accumulation and fruit yield (+22% compared to conventional PE). Particularly, the enhanced light penetration into the canopy and the reduced heat stress resulted in a significant increase in fruit number and size (Al-Madani et al., 2024).

Despite the short stature, also some leafy vegetables, like **lettuce** (*Lactuca sativa* L.) and **spinach** (*Spinacia oleracea* L.), benefit from diffusive light due to their dense canopy. Indeed, in these crops light scattering enhanced photosynthetic activity and biomass accumulation, increasing yield by 8-10% (Hemming et al., 2016). In contrast, some evidence highlighted a counterproductive effect of a PE diffusive cover on lettuce as it reduced PAR by 27% compared to control, without lowering air temperature but increasing the occurrence of tipburn (Bárcena et al., 2019). However, the increment in leaf number, avoided any negative effect on fresh and dry weight. Besides, a shade cover (lowering PAR by 76%) used as additional treatment prevented tipburn symptoms. Still in lettuce, the use of diffusive PE film showed to boost the chlorophyll synthesis, to promote a more uniform growth and to improve the health status, with a significant increase in the yield (+22% compared to conventional PE) and leaf content of total ascorbic acid (+9.4%) (Cozzolino et al., 2020).

**Tomato** (*Solanum lycopersicum* L.) is the most studied fruit vegetable under DCs and, in general, it shows a considerable improvement in photosynthetic efficiency and fruit yield when grown under a even light distribution. Indeed, this is particularly beneficial for those crops with erect habit in which the lower canopy usually faces light limitations while the upper one is exposed to light intensities above the saturation level. In these crops, diffusive covers alleviate shading of lower leaf layers and reduces photoinhibition in top leaves, while minimizing heat stress and photodamage, especially under strong sunlight conditions (Hemming et al., 2008). A PE cover with moderate diffusion properties enhanced photosynthetic activity (+21.5%) and increased tomato yield (3.2%) in Mediterranean climate, Almería, Spain (Moreno-Teruel et al., 2021). Studies conducted in the South of Italy also show that DCs boosted both early and total marketable yields of cherry tomato, with different increases depending on the rate of nitrogen fertilization (Paradiso et al., 2024). Additionally, combining DCs with a protein hydrolyzed biostimulant enhanced nutrient uptake and secondary metabolite production (i.e., phenols), improving the nutritional profile in cherry tomato (Paradiso et al., 2024). In tomato grown under a diffusive polyethylene (D-PE) film, a 15% increase in light intensity in the low canopy compared to conventional PE lead to a significantly higher photosynthetic efficiency (Moreno-Teruel et al., 2022). In tomato, highly diffusive covers can also enhanced the fruit quality and nutritional profiles, increasing lycopene, lutein, and phenolics content (+10.5%, +1.03%, and +14.5% respectively) (Ahmadi et al., 2018). Polycarbonate covers also resulted in an 8% increase in yield and improved photosynthetic efficiency in tomato (Moreno-Teruel et al., 2022).

Based on data from literature reported in **Table 1**, the highest number of crops investigated under DCs belongs to the botanical family of Solanaceae, with 19 papers in total, with 9 studies focusing on **tomato**, 2 on **pepper** and 1 on **eggplant** (*Solanum melongena* L.; Cemek et al., 2005). In these crops, the use of DCs revealed a positive impact on plant growth and yield by improving light conditions and moderating temperature fluctuations. On fruit vegetables, covers like stabilized PE-UV and PE-IR enhance photosynthesis, and increase plant growth and fruit size and yield. These effects are presumably related to the plant protection from excessive UV radiation, reducing stress conditions, and the more stable microclimate.

The findings suggest that diffusive covers, such as D-PC and D-PE, have a broad impact also on plant nutritional and metabolic responses, particularly in terms of secondary metabolite production which enhances the overall product quality. For instance, an increase in the biosynthesis of carotenoids (i.e., lycopene and lutein) was found in **tomato**, **lettuce,** and **melons**, leading to healthier plants and a better product nutraceutical profile (Ahmadi et al., 2018; Baxevanou et al., 2018).

Referring to the mechanisms underlying the plant response to diffuse light, it has been hypothesized that it enhances the growth hormone efficiency, potentially influencing hormone-driven growth responses (He et al., 2021). These effects may include improved auxin distribution, supporting shoot elongation and balanced biomass allocation.

The effect of diffuse light has been also tested in floriculture. Specifically, ***Chrysanthemums*** (*Chrysanthemum × morifolium* L.) showed to benefit from scattered light, increasing CO_2_ assimilation rate per leaf area unit (+5%), dry matter accumulation (+9.5%), number of lateral stems (+11%), and leaf area (+8%) compared with the control (Markvart et al., 2010). However, it is worth noting that the larger plant size observed under diffuse light led to a counterproductive increase in internal canopy shading compared to the direct light control.

### 4.2 Photoselective covers (PhCs)

The main properties of photoselective covers are summarized in **Figures 2** and **3**. Photoselective covers, available as colored or clear polymers, reduce the radiation reaching the crop by selectively blocking some wavelengths, altering light quality for desired physiological, phenological and morphological responses (Pandey et al., 2023). Some PhCs are designed to allow the passage of specific wavelengths, such as R to modulate the R-B-FR ratio to regulate the plant growth rate and architecture, and UV-B to stimulate the secondary metabolite production (Tafoya et al., 2018; Wong et al., 2020). Rai, 2020 showed that the increased exposure to UV (both A and B) radiation strongly induced gene expression changes in *Arabidopsis* and, in addition to promoting the plant growth, it modifies the response and interaction of various photoreceptors and alter RNA transcription. However, photoselective films experience a 2-4% reduction in light transmittance in their life span, due to weathering, and condensation can lower transmittance by an additional 5% (Abdel-Galil, 2014). Photo-induced pigment degradation within plastic matrixes can impair the cover performance, though recent materials are more durable, with some lasting up to 15 years (Blanke, 2008).

The modified light spectrum provided by photoselective films influences growth hormone regulation, promoting stem elongation and biomass accumulation (He et al., 2021). **Table 2** shows data about the effects of PhCs on various horticultural crops and how targeted growth responses, including flowering and increase of fruit size, can be attained.

**Table 2:** Effects of photoselective covers (PhCs) on plant growth, photosynthesis and secondary metabolites content in fruit, vegetable and ornamental crops.

Species	Cultivar	Material composition	Cover characteristic	Effects on plant growth, photosynthesis, secondary metabolites		References
				Increase	Decrease	
*Actinidia deliciosa* L.	Hayward	PE	PAR transmission: W (79.6%), G (72.7%), R (73.1%), B (77.2%)	W: Dry weight, soluble solids content R: vegetative vigor and carbon partitioning		Basile et al. (2012)
*Arabidopsis thaliana* L.	Wild type and mutants: uvr8-2, cry1cry2, cry1cry2uvr8-2	PE	Wavebands transmission (UV-B: 290–315 nm, UV-Asw: 315–350 nm, UV-Alw: 350–400 nm, B: 400–500 nm)	Epidermal UV screening, CHALCONE SYNTHASE transcript abundance, acclimation to drought stress		Rai (2020)
*Brassica oleracea* L.	*Capitata* F1	PP, PLA	R enhancement by 26%, reduction B and Y-G light	Biomass, WUE, photosynthesis, stress tolerance	Stomatal conductance, transpiration rate	Khramov et al. (2022)
*Capsicum annuum* L.	Ghia	Glass	Blocks most of the UV, R and FR, reducing B light transmission	Upregulation of ABA-related signaling genes (e. g., PHOT1, PHYA), ion flux in guard cells	Water use, stomatal pore size	Zhao et al. (2021)
*Citrullus lanatus* L.	Bengala	EVA	B (400–500 nm) and R light (600–700 nm) enhancement	Yield (+10%), weight, number of female flowers		Lemarié et al. (2018)
*Citrus jambhiri Lush.* L.	Kinnow Mandarin	PE	R and G nets: enhanced spectra for growth, W and S nets: light distribution, UV reduction. Stainless steel screen: heat reflection, light transmission	R and W net: plant height, budding success, N, P, Zn and Fe content	Stem diameter	Brar et al. (2020)
*Citrus sinensis × Poncirus trifoliata* L.	Daisy	Red, Green, White, Silver, and Stainless-Steel Screen Nets	R and G nets: enhanced spectra for growth, W and S nets: light distribution; UV reduction Stainless steel screen: heat reflection; light transmission	R and W: diameter, internodal sprout length, leaf area, budding success, N, P, Zn		Brar et al. (2020)
*Cucumis melo* L.	Charentais	EVA	B (400–500 nm) and R (600–700 nm) enhancement	Yield (+52%), size, weight, sugars		Lemarié et al. (2018)
	Earl’s Knight Natsukei	PE, PP	60-70% PAR transmission, 45-50% NIR absorption	Soluble solid content, brix, fructose and sucrose	Heat stress	Murakami et al. (2017)
*Cucumis sativus* L.	Aseel Hy, Safa 62	PE	UVT: UV diffusion and visible light, UVO: UV blocking	Yield (+21-25%), chlorophyll, phosphorus content	*Aphis gossypii* infestations, total phenolics, stress symptoms	Abd El-Aal et al. (2018)
	Modan	PE	10-40% light transmission	Yield (+48%), leaf area, dry weight, transpiration, stomatal conductance, CO_2_ assimilation		Tafoya et al. (2018)
*Diplotaxis tenuifolia* L.	Nature	PE	50-79 % light extinction, 64.6%-76.8% PAR reduction	Se, antioxidant activity (e. g., ascorbic acid and lipophilic, phenolic compounds)	5.93-15.01% temperature	Caruso et al. (2020)
	Reset	PMMA	Converts UV radiation into R and B, enhancing spectral quality	yield (+30%), photosynthetic efficiency, chlorophyll content, antioxidant activity, leaf greenness		Paradiso et al. (2023)
	Ramat	PE	R/FR, B/R, or B/FR ratios alternation	Yield, flowers and fruits quality	Height	Li et al. (2000)
*Eruca vesicaria* L.	Rocket	PE	27% UV-B transmission	Secondary metabolite (e. g., phenolic acids and flavonoids, luteolin and quercetin)		Mormile et al. (2019)
*Fragaria × ananassa* L.	Elsanta	PE	68-88% PAR transmission, of R/FR, B/R, or B/FR ratios alternation	Yield (+51%), flowers and fruits quality (more compact)	Petiole length	Fletcher et al. (2002)
*Lactuca sativa* L.	Kucheryavets Odesskiy	PP, PLA	R enhancement by 26%, B and Y-G light reduction	Biomass photosynthesis rates leaf area, carbon assimilation, WUE	Stomatal conductance, transpiration	Khramov et al. (2022)
	Mimosa Roxa Salad Bowel	PSF	Temperature reduction up to 1.9 °C,	Fresh and dry weight, leaf area, stem elongation, highest SPAD chlorophyll, flavonoid and anthocyanin		Amaro de Sales et al. (2021)
*Malus domestica* L.	Pinova, Fuji Kiku 8	PE	7-18% UV transmission	Fruit color intensity		Blanke (2008)
*Medicago truncatula* L.	Jemalong A17, F83005-5	PE	Wavebands transmission UV-B: 290–315 nm, UV-Asw: 315–350 nm, UV-Alw: 350–400 nm, B: 400–500 nm	Epidermal UV screening, Chalcone synthase, transcript abundance, acclimation to drought stress		Rai (2020)
*Prunus avium* L.	Lapins	PE	R/FR reduction, R, B, and R-B absorb UV and re-emit it as R, B, or both	Apical shoot growth, more vegetative activity		Schettini and Vox (2010)
*Prunus persica* L.	Messapia	PE	R/FR reduction, R, B, and R-B absorb UV and re-emit it as R, B, or both	Annual shoot growth and shoot length		Schettini and Vox (2010)
*Rubus idaeus* L.	San Rafael	EVA	Enhances B (400–500 nm) and R light (600–700 nm)	Yield (+15%), flower production, sugar content		Lemarié et al. (2018)
*Solanum lycopersicum* L.	Brenda	PE	90-100% UVA transmission, Anti NIR, heat reduction, LDe for cooler environments	Highest yield, quality and market distribution	Anti NIR and LDe provided the lowest yields due to reduced PAR and higher temperatures	Lopez Marin et al. (2019)
	Oasis and Genio	Glass	20% light transmission, dye-sensitized solar cell filters UV to enhance R and FR	Lycopene, β-carotene, antioxidant capacity	Yield, chlorophyll content, transpiration rate, stomatal conductance, photosynthetic rate	Ntinas et al. (2019)
	Unspecified	Glass, PE	NIR filters: heat reduction, and FIR filters heat retention	Yield (+3-10%)	Water use	Romero et al. (2018)
*Solanum melongena* L.	Tracey	Glass with DSSC	Filters sunlight to reduce R and B transmission while reducing overall light intensity	Flower abortion rates, total sugars	Xanthophyll pigments (e. g., antheraxanthin, zeaxanthin, violaxanthin), yield	Chavan et al. (2020)
	Ecavi	PE	3-5% UV transmission	Yield (+20%), height, leaf production, fruit quantity		Kittas et al. (2006)
*Solanum tuberosum* L.	Sirtema	EVA	B (400–500 nm) and R light (600–700 nm) enhancement	Yield (+11-13%) harvest time advanced by 8 days, small-sized tubers.		Lemarié et al. (2018)
*Triticum durum* L.	Cappelli	PE	Reduction R/FR R, B, and R- B absorb UV radiation and retransmit it in specific wavelengths (R, B, or both)	Stem height, dry weight, lateral tiller production (with differences in tiller survival between R and B)	Stomatal conductance, lateral tillers, leaf area	De Salvador et al. (2008)

The increasing or decreasing effects and the related percentage refer to results obtained in DCs compared to the corresponding clear covers.

Leafy vegetables, such as **lettuce**, show strong adaptability to spectral modifications, that enhance photosynthesis and biomass accumulation while improving water use efficiency and secondary metabolite production, hence they are suitable as advanced light manipulation technologies. In lettuce, polypropylene covers increasing R and reducing B and Y-G light significantly improved the assimilation rate and reduced stomatal conductance and transpiration, leading to a higher water use efficiency and biomass compared to non-modified cover (Khramov et al., 2022). Additionally, photoselective films enhanced both stress tolerance and produce quality in red lettuce by decreasing temperature (up to 1.9 °C), boosting leaf area, chlorophyll content, fresh and dry weight, as well as secondary metabolites, including flavonoids and anthocyanins (Amaro de Sales et al., 2021).

**Wild rocket** (cultivar ‘Reset’) showed a 30% improvement in chlorophyll content and consequently a higher photosynthetic efficiency and leaf growth and yield due to UV-to-R/B spectrum conversion (Paradiso et al., 2023). By converting harmful UV radiation, these films can improve crop resilience to sunlight-induced stress playing a pivotal role in mitigating abiotic stress, as reported by Pandey et al. (2023).

Members of Cucurbitaceae family exhibit notable responses to light manipulation strategies. **Melon** (*Cucumis melo* L.) showed a strong benefit from spectral changes in terms of productivity. Precisely, ethylene-vinyl acetate (EVA) films enhancing B and R transmission boosted yield by 52% (through both fruit size and weight) and sugar content, compared to standard film (Lemarié et al., 2018). Furthermore, NIR-absorbing PE films alleviate heat stress in melon, maintaining photosynthesis and enhancing soluble solids (Murakami et al., 2017).

These findings highlight the potential of targeted light management for optimizing fruit quality and yield. **Cucumber** plants showed a better growth under UV-transmitting covers, which increased yield by 21-25%, and enhanced leaf chlorophyll and phosphorus content. Furthermore, visible light diffusion through these covers mitigated environmental stress symptoms, leading to better plant health and reducing *Aphis gossypii* infestations (Abd El-Aal et al., 2018).

Members of the Solanaceae family, such as tomato, eggplant, and potato, show varying responses to PhCs materials and light manipulation strategies. **Tomato** grown under PE films with high UVA transmission (90-100%) and anti-NIR properties achieved higher yields and better fruit quality compared to the corresponding conventional cover. However, in some environments, anti-NIR films reduced PAR and raised temperatures, which negatively impacted yields. The PE covers with 3-5% UV transmission boosted **eggplant** yield by 20%, along with improvements in height, leaf production, and fruit quantity (Kittas et al., 2006). **Potato** (*Solanum tuberosum* L.) under EVA covers enhancing B and R transmission showed a yield increase of 11-13% (through more numerous smaller tubers) and enabled an earlier harvest (-8 days) compared to the standard EVA film without photoselective additives (Lemarié et al., 2018).

EVA copolymer-enhanced covers applied to **watermelon** (*Citrullus lanatus* L.) and **potato** improved fruit size and weight, sugar content, and yield (+10-12%), while anticipating the harvest (Lemarié et al., 2018). In **tomato**, plants under 90-100% UV-A transmission films achieved superior fruit yield and quality compared to other light-modifying technologies such as LDe (light diffusing energy) and anti-NIR covers (Lopez Marin et al., 2019). These covers can enhance secondary metabolite production, enriching the nutritional value of crops. For example, UV-B blocking films increased the phenolic acids and quercetin content in **rocket** (*Eruca vesicaria*), bolstering the plant stress resilience (Mormile et al., 2019), and high R/FR ratios enhanced the antioxidant activity in **strawberries** (*Fragaria × ananassa* L.) (Fletcher et al., 2002).

Beside the plant growth and metabolism, specific spectrum manipulation can alleviate biotic stress by controlling pathogen development and improving the plant reaction. For instance, a negative impact on fungi can be due to the higher UV-A transmission, such as in certain diffusive PEs, that may create unfavorable conditions for fungal growth and reproduction, inhibiting spore germination and mycelium development. Besides, a positive influence on the plant can depend on the enhanced light availability, improving photosynthetic efficiency, leading to stronger plant vigor, and strengthening the natural defense mechanisms and tolerance to pathogen infections. Consistently, UV-blocking films helped reducing biotic stresses, significantly lowering the occurrence of fungal diseases such as powdery mildew and early blight in **tomato** and **pepper** (Avalos-Sánchez et al., 2023).

In a trial for a new NIR-reflective film, Alsadon et al. (2016) measured a lowering of the average temperature by 9 °C compared to outside the greenhouse. The experiment also compared other commercial covers (no details on the types), which also showed a temperature decrease but of smaller magnitude (7 and 6 °C). Gas exchange parameters (photosynthetic and transpiration rate, and stomatal conductance) were associated with temperature variations, showing an inverse correlation with temperatures. The improvement in photosynthetic response resulted in a higher yield in **cucumber** (*Cucumis sativus* L.).

Red light enriched spectra stimulated tiller production and shoot growth in **wheat** (*Triticum aestivum* L.) (De Salvador et al., 2008).

Various hail nets (red, blue, grey, and white) with photoselective properties were tested on **kiwifruit** (*Actinidia chinensis* var. *deliciosa* A. Chev.), with a shading effect ranging from 20.4% to 27.3% of PAR (Basile et al., 2012). All net treatments determined an increment in light scattering and changes in light spectrum and influenced productive traits: red and blue nets increased fruit weight and dry matter content, while grey and white nets improved fruit firmness and reduced fruit drop, indicating that net color can be used to modulate both fruit yield and quality. However, different response to shading and spectral changes were observed in the two years of observation, highlighting the occurrence of the interaction of treatments with climate conditions.

On plants of the genus ***Prunus*** (*P. avium* L. and *P. persica* L.) grown in pots, various plastic photoselective and photoluminescent films were tested, revealing that altering the spectrum (mainly R and FR) affected the vegetative activity and photomorphogenesis of cherry and peach shoot, and highlighting how the use of B photoselective films is useful in containing the plant size while R and G covers to increase it (Schettini and Vox, 2010).

In **lemon** (*Citrus jambhiri* L.), red nets help mitigate thermal stress by diffusing solar radiation, which reduces excessive heat buildup and enhances nutrient content and growth (Brar et al., 2020).

In the ornamental plant ***Anthurium andraeanum***, PE, NIR filters increase the flower stem yields by 3-10% (Romero et al., 2018). In **chrysanthemum** (*Dendranthema grandiflorum* L.), R-FR spectrum enhanced plant height and flower quality (Li et al., 2000).

#### 4.2.1 Luminescent photoselective covers (LPhCs)

The luminescent photoselective covers include the three primary classes of materials used in luminescent covers: organic dye molecules, quantum dots, and rare earth ions.

Organic-Based Dyes in Luminescent Covers (ODLC), especially polycyclic aromatic hydrocarbons (PAHs), offer significant potential for LPhCs due to their high fluorescence quantum yields and affordability. These dyes absorb light at specific wavelengths (efficiently shifting photons with energies above their bandgap) and convert it into usable energy, while light at other wavelengths either passes through the material or is dissipated as heat. PAHs, such as perylene derivatives, excel in absorbing G light, which is less efficient for photosynthesis than B or R, making them advantageous for the agricultural use (Banal et al., 2017).

Quantum Dots (QDs) in LPhCs exhibit fluorescence in colors that vary with the particle size, with smaller crystals emitting shorter wavelengths. This size-dependent tunability enables the precise control over the light spectrum emission (Resch-Genger et al., 2008). In greenhouse applications, QDs embedded in films transform UV and B into R radiation that better support photosynthesis (Makarov et al., 2019). Compared to organic dyes such as PAHs, QDs offer a greater separation between absorbance and emission spectra, minimizing reabsorption and improving light efficiency (Makarov et al., 2019). Nonetheless, practical challenges persist in modulating QDs emission to match the plant requirement and optimize growth, as environmental variables like sunlight intensity and geographical location can influence their effectiveness (Shen and Yin, 2022).

These innovative LPhCs enhance photosynthetic efficiency, significantly benefiting leafy greens in growing conditions with limited PAR, by improving plant physiological traits and enhancing pigment concentrations and overall leaf health. In **wild rocket** (*Diplotaxis tenuifolia* L.), photoluminescent films promoted photosynthetic pigment concentrations (Paradiso et al., 2023).

Dye-Sensitized Solar Cell (DSSC) integrated glass reduced light intensity, mitigating flower abortion while boosting xanthophyll pigments such as zeaxanthin and violaxanthin in **eggplant** (Chavan et al., 2020). Advanced glass covers with DSSC filters (UV- and FR- blocking) improved chlorophyll content and photosynthetic rates, and enhanced lycopene, β-carotene, total carotenoids and antioxidant capacities in **tomato** cultivars (Ntinas et al., 2019). In contrast, the use of these covers on **pepper** (*Capsicum annuum* L.) reduced the stomata size and abscisic acid production, resulting in a faster stomatal response to light changes, and compromising water use efficiency (Zhao et al., 2021). Similarly, **cabbage** (*Brassica oleracea* L.) showed higher photosynthetic rate under increased R and reduced B and Y-G wavelengths (Khramov et al., 2022).

High quantum efficiency and durability of materials are priority features in designing luminescent solar concentrators (LSCs) (Griffini et al., 2013). Integrating photovoltaic (PV) technologies into greenhouse settings has frequently led to yield reductions (Cossu et al., 2016; Loik et al., 2017), although spectral-shifting covers using perylene and QDs were proven to improve the productive crop potential. However, cost-efficiency often outweighs high-performance requisites in horticulture. This balance between cost and crop productivity continues to drive innovation in greenhouse technologies.

### 4.3 Switchable covers (SCs)

The main properties of switchable covers are summarized in **Figures 2** and **3**. The optimal characteristics of a greenhouse cover vary according to the geographic location and crop type, as no single cover material is universally suited to all crop-climate combinations. Typically, greenhouse covers have fixed optical properties that regulate the amount of sunlight entering the structure. As a result, the intensity and quality of sunlight (including spectrum and balance between direct and diffused light) may not be ideal for the crop throughout its growth cycle, since light requirements change in the developmental stages (Baeza et al., 2019). To address these limitations, new materials with switchable optical properties are being developed, allowing nearly instant adjustments to light conditions inside the greenhouse or employing supplementary methods like temporary coatings, mobile/fixed screens, and heating/cooling systems (Baeza et al., 2019).

Switchable covers (SCs) are dynamic materials that adjust their features in response to environmental changes, such as temperature or light intensity, offering greater flexibility in regulating light and thermal parameters during the different plant developmental stages. These covers dynamically filter PAR and NIR light, optimizing light quality to improve photosynthesis. For instance, electrochromic and thermochromic covers adjust transparency in response to environmental triggers, optimizing the growth conditions in greenhouse. This technology reduces the heat stress while enhancing photosynthetic efficiency, ensuring consistent growth even in extreme conditions (Baeza et al., 2019, 2020). Many PhCs and SCs are engineered with UV stabilizers and weather-resistant polymers to extend lifespan up to 10–15 years (e.g., Blanke, 2008; Baeza et al., 2020).

Effects of SCs on plant growth, photosynthesis and secondary metabolism in vegetables and ornamentals are shown in **Table 3**.

**Table 3:** Effects of switchable covers (SCs) on plant growth, photosynthesis and secondary metabolites content in vegetable and ornamental crops.

Species	Cultivar	Material composition	Cover characteristic	Effects on plant growth, photosynthesis, secondary metabolites		References
				Increase	Decrease	
*Anthurium andraeanum* L.	Unspecified	Thermochromic glass	Switchable FIR filter (65% reduction)	Yield (+3-10%) Estimated data (Modelling study)	NIR-selective filters: water use	Romero et al. (2018)
*Cucumis melo* L.	Unspecified	Thermochromic PC	Passive heating during cold season, cooling effect at high temperatures	Early flowering, stable, sugar content	Heat stress	Pandey et al. (2023)
*Solanum lycopersicum* L.	Unspecified	Electrochromic glass	NIR selective filters, TIR filters dynamic control of light transmission (NIR, PAR, TIR), change properties based on temperature (28°-30 °C)	Yield (+12-22%), fruit quality, microclimate control	Energy and resource use, disease risk	Baeza et al. (2019)
	Unspecified	Thermochromic polymer: VO_2_ Photochromic polymer: SPs, DAEs Electrochromic polymer: PANI, PEDOT	PAR + NIR filters; dynamic adaptation to high solar radiation; automatic shading effect in response to temperature (26°-32 °C)	Yield (+15%) Estimated data (Modelling study)	Water consumption, supra-optimal temperature exposure	Baeza et al. (2020)
	Unspecified	Thermochromic PE	Switchable NIR filter (10% PAR reduction), 28°-30 °C	Yield (+15-20%)	Potential winter performance	Romero et al. (2018)
	Unspecified	Thermochromic PE: (OPV) Review article	Dynamically adjusts light transmission based on sunlight intensity	Yield (+19%), earlier harvest, fruit quality, WUE	Heat stress	Soussi et al. (2022)
*Solanum tuberosum* L.	Unspecified	Thermochromic laminated PE	Adaptive light control, extreme heat reduction	Growth, earlier harvest	Risk of temperature-related stress	Pandey et al. (2023)
*Rubus idaeus* L.	Unspecified	Thermochromic glass with IR filtering	Dynamic shading effect, temperature fluctuations reduction	Fruit quality, better sugar accumulation	Excessive shading effects	Pandey et al. (2023)

The increasing or decreasing effects and the related percentage refer to results obtained in DCs compared to the corresponding clear covers.

In Mediterranean climates, these covers can significantly improve light and temperature conditions, especially in regions with fluctuating climate (Romero et al., 2018; Soussi et al., 2022). Indeed, SCs stabilize physiological processes by preventing excessive transpiration and reducing stress-induced metabolic changes. It is modelled that this regulation promotes crop health in high-value vegetables like **tomato** and ornamentals like ***Anthurium andraeanum***, ensuring stable growth even under challenging environmental conditions (Romero et al., 2018; Soussi et al., 2022). It is estimated that under SCs **tomato** yield can increase up to 20% due to the improved environmental conditions (Romero et al., 2018; Baeza et al., 2019). Additionally, by stabilizing temperature and minimizing stress responses, SCs have an indirect positive influence on plant hormonal balance. This is particularly beneficial for sensitive crops like **tomato**, where hormonal disruptions can negatively impact plant productivity (Romero et al., 2018).

For fruit crops, in **melons**, SCs promoted early flowering and increased sugar content while mitigating heat stress during high temperature periods (Pandey et al., 2023). Dynamic shading improved fruit quality and sugar accumulation in **raspberry** (*Rubus idaeus*), though excessive shading remains a potential drawback (Pandey et al., 2023).

In tuber crops like **potato** (*Solanum tuberosum* L.), thermochromic covers help regulate temperature, reducing extreme heat exposure and promoting growth and earlier harvest (Pandey et al., 2023).

Overall, the efficiency of SCs varies across the crop types: to summarize, high-value ornamentals benefit from stabilized physiological processes, root and tuber crops experience enhanced early growth, and fruit vegetables show improvements in yield, fruit quality, and stress tolerance. However, SCs application is influenced by several other factors, such as initial and maintenance costs, crop productivity goals, local climate, and technological support (Ahmadi et al., 2018).

### 4.4 Control technologies and technological relevance

**Passive dynamic control technologies** represent a significant advancement in greenhouse management, as they automatically adapt their properties in response to environmental changes, without requiring external energy input. These technologies allow materials to react to varying climate parameters such as temperature, sunlight, and humidity, thereby enhancing the energy efficiency while maintaining better growth conditions in greenhouse (Soussi et al., 2022; Zhang et al., 2022; Ghiasi et al., 2023). For instance, thermochromic materials alter their properties based on temperature fluctuations, effectively reducing heat gain and providing thermal comfort for the plants (Padilla et al., 2021). Similarly, photochromic materials adjust their transparency in response to light levels, minimizing the glare (Nikolaou et al., 2018; Lin et al., 2022). Additionally, photoelectric materials, including crystalline and inorganic thin-film glazing, not only provide shading to protect plants from excessive sunlight but also generate energy, further contributing to the sustainability of greenhouse operations (Timmermans et al., 2020; Maraveas et al., 2021, 2023a).

to modify their properties, facilitating real- By integrating these innovative materials, greenhouse managers can optimize growing conditions while saving resources.

**Active dynamic control technologies** require an external energy source time adjustments tailored to specific environmental conditions (Baeza et al., 2020). These systems provide a precise control over light transmission and diffusion, which can significantly enhance plant growth and energy efficiency. For instance, electrochromic materials can change their opacity or color in response to an applied electric current, allowing for meticulous control over both light and heat transmission (Baeza et al., 2020; Maraveas et al., 2023a). On the other hand, mechano-chromic materials adjust their properties through mechanical inputs, such as deformation, which further enhances their adaptation to varying environmental scenarios (Guo et al., 2024). Additionally, polarized particle devices, including Suspended Particle Devices (SPDs) and Polymer Dispersed Liquid Crystals (PDLCs), are controlled via electrical signals to effectively modulate light and heat in the greenhouse (Ghosh et al., 2024). By incorporating these advanced technologies, greenhouse operators can optimize growing conditions dynamically, to improve crop yield and resource management.

To improve plastic properties, like temperature resistance, heat dissipation, droplet formation and dust deposition prevention, some functional additives, fillers, air bubbles, reinforcements (e.g., glass or carbon fiber), and colorants are often incorporated Castilla (2013). Examples are UV absorbers and stabilizers that help protect plants in greenhouse while blocking UV-B radiation over 40 kJ/m², controlling the UV transmission rate, typically 70-90% in common materials (Zhang et al., 2019). Anti-fog and IR-blocking additives further prevent fogging and restrict harmful IR radiation (Kittas et al., 2006).

To protect plastic films and panels from UV damage and photodegradation, stabilizers and additives like black carbon modify the optical properties of cladding materials (Aldaftari et al., 2019). The UV absorbers and additives also shield plants from harmful radiation and help limit the spread of pests and pathogens (Antón et al., 2014).

Infrared light absorbers minimize heat loss and short-wave emission, typically having wavelengths between 700 and 2500 nm. Long-wave absorbers (2500-40,000 nm) reduce heat loss from plants in greenhouse by absorbing these wavelengths. Surfactants and antistatic agents lower surface tension to reduce the dust accumulation on plastic films (Maraveas et al., 2023b).

Red-emitting plastic greenhouse extensions provide stunning and vivid patterns and colors. As the pigment volume fraction increases, the efficiency of greenhouse gases rises. For optimal radiation control, HVAC (heating, ventilation, and air conditioning) systems are generally more effective than pigments, as titanium dioxide (TiO_2_) and diamond particles in pigments can selectively reflect near-IR (800–2500 nm) while transmitting visible light at shorter wavelengths (Aldaftari et al., 2019).

Diamond-based pigments are advanced materials used in greenhouse cladding to optimize light management and thermal control. They effectively reduce heat buildup while allowing sufficient light for photosynthesis, offering a cost-efficient alternative to traditional cooling systems. Their unique optical properties enable them to reflect near-IR radiation while transmitting visible light, surpassing conventional pigments like TiO_2_ in performance. Additionally, TiO_2_ particles are less effective in reflecting IR radiation, and diamond particle-based pigments have been shown to reduce radiation (Aldaftari et al., 2019). Transparent Solar Distillers (TSD) combine TiO_2_ nanoparticles in multi-purpose greenhouse coverings to use solar energy for water desalination (Rabhy et al., 2019). These TSD pigments also enhance greenhouse cladding by managing incoming and outgoing energy wavelengths. By filtering high-energy wavelengths during the day, greenhouse covers prevent overheating while ensuring sufficient light for photosynthesis. At night, they retain heat by limiting long-wavelength energy loss, supporting stable internal temperatures for crop growth and development.

One of the more recent innovations involves the use of antimony tin oxide (ATO) nanoparticles in plastic films, which help manage solar radiation while maintaining adequate light levels for photosynthesis. These nanoparticles reduce the amount of heat absorbed by the greenhouse while allowing enough PAR to pass through, ensuring both energy efficiency and healthy crop growth (Zhang et al., 2024a).

## 5 Discussion and conclusions

The reviewed studies highlight the importance of selecting the appropriate greenhouse cover based on the specific climatic conditions, crop type, and desired outcomes. Smart covers support sustainable greenhouse farming by regulating light and temperature, reducing the need for artificial lighting, heating and cooling, thereby lowering the overall energy use. Indeed, advanced materials are durable and weather-resistant, adjust transparency and minimize temperature and humidity fluctuations, enhancing plant performance, and maximizing the resource use efficiency. Besides, they filter UV rays, reducing pest diffusion and limiting chemical treatments.

It is worth noting that no cover type is universally superior and each one shows strengths and limitations and can fully express its potential in different crop-environment conditions.

**Diffusive covers** improve light uniformity in the canopy profile, increasing plant photosynthesis and crop yield, especially in high-light environments. They are universally applicable across climates and crop types and are low-maintenance solutions. On the downside, they can reduce PAR, leading to disorders in light-sensitive plants.

**Photoselective covers** determine changes in light spectrum, increasing plant growth and promoting modulation of target photomorphogenic responses (e.g., flowering, secondary metabolites biosynthesis), while reducing heat stress. Additionally, they can help alleviate pathogen pressure (via UV filtering) and microclimate adjustments (via NIR reflection). However, optimizing one wavelength can be disadvantageous to another and lead to spectrum imbalance and the increase in nutritional quality can lead to a decrease in yield. Besides, they do not allow spectrum manipulation through the crop phenological stages.

**Luminescent Photoselective Covers** convert less useful UV radiation into photosynthetically active radiation, boosting pigment and antioxidant levels and enhancing photosynthetic performance under low-light conditions. In addition, they allow spectrum changes when weather conditions change. On the other hand, latitude impacts their reliability therefore careful system calibration is needed to avoid spectrum imbalance.

**Switchable covers** dynamically adjust light transmission in PAR and NIR wavebands in response to environmental cues, enabling seasonal photothermal control, which improve crop performance. They offer flexibility and energy efficiency and are ideal for regions with fluctuating weather conditions. They overcome the limits of photoselective covers, allowing adjustment of light and thermal environment according to the plant requirement in the different phenological stages. Clearly this sensitivity, together with the need for frequent and efficient sensors calibration, turns out to be higher initial cost and maintenance.

To summarize, the simpler technologies (DC, PhC) offer benefits with lower technical requirements and cost, while more advanced technologies (LPhC, SC) allow specific and dynamic responses but need higher technical specialization of farmers and are more expensive. However, they are not mutually exclusive, hence future innovation in the greenhouse industry could rely on hybrid solutions, combining different technologies.

In conclusion, the adoption of innovative smart covers can provide an effective tool to enhance the produce yield and quality while reducing the greenhouse energy cost and environmental impact, but two remarks are needed. The first is that the improvement in biochemical traits (like vitamins and antioxidants) may not always translate into a greater economic gain for farmers; the second concerns the environmental impact of the cover disposal.

Future research should continue to optimize the spectral properties of greenhouse covers for specific crops and climates, to achieve the best possible balance between productivity and quality, and sustainability. Specifically, it should focus on the characterization of response of the different crops (particularly in high-value and specialty plants) and the fine-tuning of spectral and thermal properties of covers to meet the specific crop and climate requirements. Integrating smart technologies such as advanced sensors and automated control systems could allow for dynamic adjustments of the greenhouse environment to optimize growth conditions. Research should also prioritize the development of recyclable and biodegradable cost-effective materials to improve their environmental and economic sustainability. On these bases, collaboration between material experts, agronomists, and greenhouse engineers will be crucial in achieving breakthroughs that meet both economic and environmental needs.
